# COVID-19 Symptoms and Mental Health Outcomes among Italian Healthcare Workers: A Latent Class Analysis

**DOI:** 10.3390/healthcare12141403

**Published:** 2024-07-15

**Authors:** Giulia Foti, Luca Merlo, Georgia Libera Finstad, Gabriele Giorgi

**Affiliations:** Department of Human Sciences, European University of Rome, 00163 Rome, Italy; luca.merlo@unier.it (L.M.); georgialibera.finstad@unier.it (G.L.F.); gabriele.giorgi@unier.it (G.G.)

**Keywords:** COVID-19, healthcare workers, mental health, post-traumatic stress, LCA

## Abstract

The COVID-19 pandemic has led to long-lasting consequences for workers leading to what has been termed a “psychological pandemic”. Some categories, such as healthcare workers (HCWs), are considered high risk due to factors such as increased exposure and stressful working conditions. In this study, we investigate whether levels of posttraumatic stress symptoms and COVID-19-related fear (IES-6 and PSI-4) are associated with illness severity in a sample of 318 infected HCWs in Italy. To investigate the presence of different profiles of COVID-19 severity, Latent Class Analysis (LCA) was performed based on 11 symptoms. Differences in the IES-6 and PSI-4 scores across the latent classes were compared using the non-parametric Kruskal–Wallis (KW) test with Dunn’s multiple comparison post hoc testing. Our analyses show that the LCA identified three classes of symptoms, reflecting no/low, mild and severe symptoms. The classes include vomiting, confusion, conjunctivitis, diarrhea, dyspnea, headache, ageusia, fever, anosmia, osteo muscle articular pain and asthenia. We found that HCWs who experienced more intense symptoms reported significantly higher IES-6 and PSI-4 scores. Moreover, we found gender-related differences in IES-6 and PSI-4 scores as females exhibited higher levels than males. Indeed, these findings are useful for developing health prevention and emergency management programs.

## 1. Introduction

### 1.1. COVID-19 Pandemic: The Symptoms and the Traumatic Effect of the Disease

The Coronavirus disease 2019 (COVID-19) pandemic has had a significant effect on the lives of people and communities around the world and continues to have it nowadays. Although the pandemic is no longer considered an emergency, it is essential to analyze its psychophysical consequences to develop future prevention plans. Indeed, one of the critical factors consists of being able to intervene promptly and adequately to reduce both the threats to physical health (e.g., risk of infection) and the psychological and social effects that contribute to decreasing the well-being of individuals. Regarding disease progression, after transmission, symptoms of infection occurred most frequently 4 and 5 days after exposure [1]. According to a study conducted in China [2] the most frequent symptoms were fever, cough, fatigue, sputum production, shortness of breath, myalgia or arthralgia, sore throat, headache, chills, nausea or vomiting, nasal congestion, and diarrhea. Asymptomatic infection mainly concerns young patients aged 18–29 years [3]. The severity of the infection ranged from mild to severe and even fatal. The course of the disease can be progressive, in fact over the course of a week the symptomatology can become mild to moderate or severe. The average time to onset of dyspnea was 5 days, to hospitalization 7 days and to the development of Acute Respiratory Distress Syndrome Symptoms (ARDS) was 8 days from the onset of the disease [3]. The COVID-19 disease involves not only physical symptoms but also psychological effects which have led to detrimental consequences for the mental health of the population. In this regard, the literature highlights how the most frequent psychological effects have been associated with anxiety, stress and depression [4]. The unpleasant experience of contagion or its suspicion exposes the individual to the risk of developing further psychosomatic symptoms, insomnia, anxiety, feelings of loneliness and depression, corroborating the negative impact of the pandemic on mental health. Experiencing insecurity about one’s health status (e.g., suspected infected cases) would increase the development of anxiety symptoms and obsessive–compulsive behaviors [1]. Indeed, fear of infection generates high levels of anxiety [5] also associated with the consequences that could result from contracting the virus [6]. Therefore, following the definition of traumatic experience as any event that creates a condition of insecurity and disorder in the individual or in society, shattering his/her beliefs and expectations that he/she builds about the world [7], the global COVID-19 pandemic can be analyzed as a type of traumatic stressor. In this framework, the results of the research conducted by Bridgland and colleagues [8] showed that participants presented symptoms similar to post-traumatic stress disorder (PTSD) both due to events that did not happen and when they had been directly exposed to the virus and indirectly through exposure to the media. In fact, 13.2% of the participants presented post-traumatic symptoms even though the types of exposure to COVID-19 did not fall within diagnostic criteria. In addition, the emotional impact of the worst experienced/expected events has been found to be a predictor of post-traumatic stress disorder (Bridgland et al., 2021). As reported by Sanchez-Gomez and colleagues [9], the pandemic induced individuals to experience real intrusive thoughts that fueled states of COVID-19-related fear. More specifically, the authors showed that hyperarousal played a crucial mediating role in this relationship. Hence, the presence of posttraumatic stress symptoms has been widely described and highlighted within the pandemic context [10,11].

### 1.2. Theoretical Background and Hypothesis Development

#### The Effect of the COVID-19 Pandemic on Healthcare Workers

During the pandemic, the primary problem that affected HCWs was the risk of being infected, due to several factors, such as increased exposure and long working hours [12]. As described above, this virus carries a variety of symptoms and different grades of severity. Some studies in the literature have focused on the classification of physical symptoms of COVID-19 into subgroups. For example, a study by Ferrat [13] and colleagues identified six symptom classes: “paucisymptomatic”, “anosmia and/or ageusia”, “influenza-like syndrome with anosmia and ageusia”, “influenza-like syndrome without anosmia or ageusia”, “influenza-like syndrome with respiratory impairment”, and a “complete form”. Similarly, six classes were identified in a study by Silveira Moreira [14], which emphasized distinctions between (1) all the symptoms, (2) high prevalence of symptoms, (3) predominance of fever, (4) predominance of cough/sore throat, (5) mild symptoms with predominance of headache, and (6) absence of symptoms. Additionally, an Iranian study [15] classified infected individuals into three distinct classes: mild disease, moderate disease, and severe disease which involve a high probability of having cough, shortness of breath, and sore throat. These studies contribute to a better understanding of the different groups of symptoms and their combinations, providing a detailed and heterogeneous view of the disease’s manifestation. Furthermore, due to the risk of contracting the virus, HCWs were more likely to experience psychological effects and greater psychosocial difficulties such as drowsiness, anxiety, depression, somatization and obsessive–compulsive symptoms compared to non-healthcare workers [12]. Indeed, there is a significant positive association between fear of COVID-19 with (a) workplace panic anxiety and (b) workplace avoidance behavior. Physicians with significant fear levels reported higher levels of workplace panic anxiety and avoidance behavior [16]. Furthermore, a study conducted on a sample of 1379 Italian HCWs [10] showed that 49.38% of respondents reported post-traumatic symptoms, 24.73% exhibited symptoms of depression, 24.73%, 19.80% indicated anxiety symptoms, 8.27% exhibited problems of insomnia, and finally, 21.90% reported high perceived stress. Furthermore, as underlined by a large body of evidence, numerous healthcare workers have been infected by COVID-19, being more exposed to contagion. Indeed, the severity of infection and also fear of death can lead to perceiving a traumatic event and developing PTSD [17]. In this regard, healthcare workers who have contracted COVID-19 have been found to have significantly higher levels of depression, anxiety and stress than non-infected workers. Specifically, higher levels of anxiety, stress, depression, intrusion, hypervigilance and avoidance were recorded. In fact, as this category of patients was exposed to greater initial stress related to the high risk of infection, they presented more negative psychological responses compared to non-infected workers [18,19]. Indeed, in accordance with the Conservation of Resources (COR) theory [20], individuals who already start with few personal resources are less likely to acquire new ones, and therefore the ability to cope with stressful situations may be impaired. Furthermore, in line with the second corollary of the theory, stress can occur when there is a loss of resources; the impact of this loss is perceived by individuals as more significant than the gain’s impact. This can also lead to cascading loss effects [21]. The presence of physical symptoms due to infection can compromise the health of the individual, thus contributing to the loss of resources and determining a greater individual vulnerability to the onset of psychological consequences such as anxiety and PTSD. Indeed, loss of resources may lead to psychological distress [21]. It is precisely PTSD that occurs when the individual experiences a significant loss of resources that undermines survival [20,22]. In line with the COR theory principles, when the individual experiences a threat to their safety, understood as an attack on their health, this translates into a traumatic response [22]. Moreover, the perceived threat can vary based on the symptomatology, leading to different mental health responses. Having more resources can facilitate the acquisition of additional resources, while a loss of resources can trigger a cycle of further loss [20]. A severe symptomatic presentation may cause a more significant loss of resources compared to a less severe presentation, as the individual perceives an inability to cope with the severity of the situation, resulting in a greater impact on mental health.

HCWs are consistently exposed to a significant risk to their health, which could have adverse effects on their physical and psychological well-being. A large body of studies in the literature (e.g., [18,19]) have examined the relationship between physical symptoms and negative psychological consequences in this population. This investigation can represent a significant contribution to existing scientific knowledge, especially for HCWs who are at high risk of exposure to physical illness or epidemic, such as COVID-19, and who also require psychological recovery to facilitate return to work and provide effective care.

In addition, this study uses a person-centered approach to classify physical symptoms and to evaluate different levels of mental health, examining the differences between individuals in depth. The current research design aims to use a cluster analysis technique to investigate possible distinctions among healthcare victims of COVID-19 with psychological consequences or highlight different degrees of mental health. By not looking at total scores but examining different classifications, we can establish not only a dichotomous distinction between healthy and unhealthy but also highlight different gradations of mental health. Literature studies have focused on how phenomena such as burnout should not be considered as a polarized concept, but rather distinct patterns or profiles may exist within the organizational context and identifying them could enhance understanding of workers’ experiences and distress [23,24]. In line with this, the use of the LCA technique has been employed to understand the possible configurations that COVID-19 has generated on mental health [25]. Understanding the presence of these possible heterogeneous configurations through the use of COR theory also allows us to shed light on how individuals may acquire or lose resources resulting from the presence of such a traumatic event as the COVID-19 pandemic and how this may affect their well-being. In this regard, there are studies aimed at developing and validating a scale that applies COR to COVID-19 [26]. To explore the existence of different classes of post-COVID-19 symptomatology severity, we used Latent Class Analysis (LCA) [27]. Using LCA allows us to identify different symptom profiles and group individuals with similar patterns of COVID-19 symptoms. Then, we leveraged the obtained latent class variable to examine differences between COVID-19-related PTSD, and COVID-19 relation between groups of healthcare workers using Kruskal–Wallis (KW) tests and Dunn’s multiple comparison post hoc analyses.

Formally, we aim to evaluate the following hypotheses:

**Hypothesis 1 (H_1_):**  *Different COVID-19 symptom classes can be identified among HCWs.*

**Hypothesis 2 (H_2_):**  *There are differences in levels of COVID-19-related PTSD and fear of COVID-19 between the identified subgroups.*

Furthermore, the transactional theory of stress and coping by Lazarus and Folkman [28] provides an effective lens through which to view stress as an interaction between the individual and their environment, as well as the individual’s perception of a discrepancy between their capabilities and the demands of their environment. The theory places significant emphasis on the cognitive appraisal phase distinguished by primary and secondary appraisal and on the strategies that people use to manage stressful internal and external demands of situations, defined as coping [28]. Indeed, the onset of COVID-19 physical symptoms can be evaluated, during the primary appraisal, as a severe threat to personal health. Subsequently, during the secondary appraisal, the individual may evaluate his or her personal resources as insufficient to cope with the threat of symptoms, generating a distress reaction. Specifically, we also assume that the effects on mental health inducted by COVID-19 severity symptoms may vary by gender. As shown by Fyers and colleagues [29], in fact, women exhibit a higher level of psychological distress than men. A large body of research has focused on the significant impact that cognitive factors have on the traumatic response [30,31,32] considering the moment of cognitive evaluation capable of generating different traumatic responses. In fact, women are more likely to report assessments of threats and losses than men [33,34] and are significantly more likely to evaluate events as stressful and to report higher perceived distress in terms of loss of control and lack of alternative coping strategies [35]. Women are more at risk for PTSD [36] apparently because they have a greater loss of appraisal control than men [37]. In addition, regarding the subjective evaluation of stress, it has been shown that women pick up threat signals more easily than men [38]. Based on this framework, in this study, we investigate whether levels of PTSD symptoms and COVID-19-related fear are associated with disease severity in a sample of Italian healthcare workers. We leveraged the obtained latent class variable to examine differences between COVID-19-related PTSD, and COVID-19-related groups of healthcare workers classified by gender using Kruskal–Wallis (KW) tests and Dunn’s multiple comparison post hoc analyses.

Therefore, we propose our final hypothesis:

**Hypothesis 3 (H_3_):**  *There are gender-based differences in mental health outcomes associated with increased COVID-19 symptom severity among HCWs.*

## 2. Materials and Methods

### 2.1. Study Design

We retrospectively collected data on health care professionals working in a tertiary referral hospital employing approximately 5000 HCWs in Florence, Tuscany, Italy. Between 1 October 2020 and 30 April 2021, 440 were infected with COVID-19 and tested positive for SARS-CoV-2 infection via an RT-PCR nasopharyngeal swab (Real-Time Polymerase Chain Reaction). After the acute phase of the illness, all the workers were contacted and invited to a post-COVID-19 return-to-work visit at the Occupational Medicine Unit. Of the 440 workers who tested positive in the considered period, 318 accepted the invitation for the medical examination, constituting a participation rate of 72.3%. We considered HCWs as all workers involved in the care of patients. The administered questionnaire included pandemic-related distress, mental health-related risk factors and sociodemographic characteristics of the respondents. The study was conducted in accordance with the Declaration of Helsinki, and all participants gave their informed consent. Given the observational nature of the study, and in the absence of any involvement of therapeutic medication, no formal approval of the Institutional Review Board of the local Ethics Committee was required.

### 2.2. Measurements

As COVID-19 factors, we gathered information on 11 self-reported infection symptoms, namely vomiting, confusion, conjunctivitis, diarrhea, dyspnea, headache, ageusia, fever, anosmia, osteo muscle articular pain and asthenia.

COVID-19-related post-traumatic stress was measured using the 6-item version of the Impact of Event Scale (IES-6) [39] rated on a 5-point Likert scale. The scale used COVID-19 as the specific traumatic event. Specifically, the IES-6 includes 2 items for each of the post-traumatic stress dimension: intrusion (e.g., “Since the beginning of the COVID-19 emergency, I thought about it when I didn’t mean to”), avoidance (e.g., “Since the beginning of the COVID-19 emergency, I was aware that I still had a lot of feelings about it, but I didn’t deal with them”), and hyperarousal (e.g., “Since the beginning of the COVID-19 emergency, I had trouble concentrating”). A total score is calculated by obtaining the sum of all six items, ranging from 6 to 30. Higher scores correspond to greater PTSD symptoms. Cronbach’s alpha had a value of 0.86 with an associated 95% Confidence Interval (CI), 0.84–0.89.

COVID-19-related fear was measured using the PSI-4 scale ([9], p. 202) evaluated on a 5-point Likert scale. The specific items about the experience of fear were the following: “How afraid were you of getting infected with the SARS-CoV-2/COVID-19?”, “How afraid were you of being quarantined?”, “How afraid were you that the infection could worsen pre-existing conditions?” and “How scared were you of transmitting the infection to your family members?”. Cronbach’s alpha had a value of 0.68 with an associated 95% CI, 0.61–0.73. A total score has also been calculated by summing up each item score, ranging from 4 to 20. The higher the score, the greater the fear of COVID-19. For both sets of questionnaires, IES-6 and PSI-4, the possible answers were formulated as “Not at all”, “Slightly”, “Moderately”, “Very” and “Extremely”. The minimum score possible for each question is 1, and the maximum is 5.

Finally, sociodemographic characteristics were sex (0 = male, 1 = female), age (years), Body Mass Index (BMI, kg/m^2^), job type within the healthcare sector as a five-level categorical variable: physicians (structured doctors), nurses, healthcare assistants (workers in support of nurses and doctors, who assist patients with daily personal hygiene activities and carry out simple activities to aid nursing and technical healthcare activities), resident physicians (doctors in training), and others (radiology technicians, lab technicians, physiotherapists, midwives, and patient transport workers). Lastly, the respondents were classified as frontline workers defined as participants who reported direct patient contact; meanwhile, those who did not report direct patient contacts were classified as non-frontline.

### 2.3. Statistical Analyses

As a preliminary investigation, a descriptive analysis was performed to examine the distribution of the variables in the entire sample and across sex. Continuous variables are expressed as mean and standard deviation (in parenthesis) while categorical variables are reported as absolute frequency and percentage frequency (in parenthesis). Associations between categorical variables were assessed by Fisher’s exact test; for normally distributed continuous variables, differences between means from two groups were evaluated using the two-sample *t*-test otherwise the non-parametric Wilcoxon test was used for non-normally distributed variables. The normality assumption was tested using an extension of the Shapiro–Wilk’s (SW) test.

**Figure 1:**
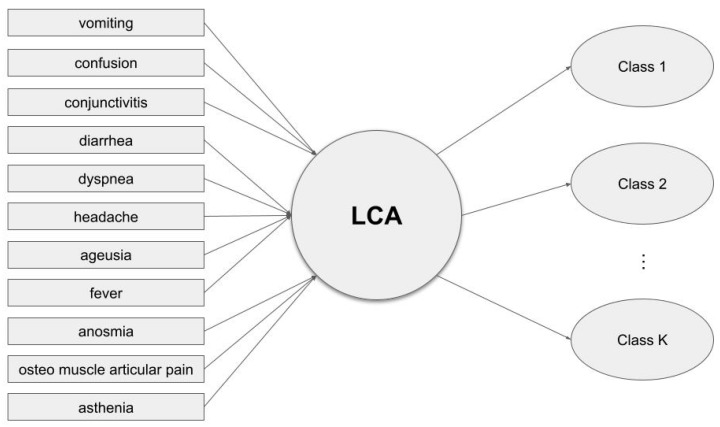
Representation of the considered LCA model. Note: Rectangles show the manifest variables while ellipses represent the latent classes determined by the LCA.

In the first step of the analysis, to determine the existence of different degrees of infection severity (Hypothesis H_1_), an LCA was fitted on the considered 11 COVID-19 symptoms from all the survey participants. LCA is a well-known statistical procedure that aims to find groups, also known as classes, that cannot be observed directly, sharing common observable characteristics within a population of interest. To identify such classes, LCA uses categorical variables collected from the study participants to reveal natural subgroups with high within-class homogeneity and high between-class heterogeneity, without imposing stringent constraints on their composition. In our case study, the LCA was performed to determine distinct subgroups of HCWs identified by symptom occurrence, which is essential for the identification of individuals at risk (Figure 1).

To identify the optimal number of latent classes, we repeated the LCA for different numbers of classes from 1 to 10 and retained the best solution corresponding to the lowest Bayesian information criterion (BIC) value. All models were estimated using Maximum Likelihood estimation. As a sensitivity analysis, although not used to select the final model, we examined additional diagnostic criteria: Akaike Information Criterion (AIC), Approximate Weight of Evidence Criterion (AWE), Consistent Akaike Information Criterion (CAIC) and the Sample-size Adjusted Bayesian Information Criterion (SABIC). After we identified the best class model, we then assigned each individual to a specific class based on its highest posterior class membership probability, that is, the probability of a unit being assigned to a class given the scores of the variables used to create the classes.

In the second step of the analysis, we conducted multiple group analyses to examine differences in depressive and anxiety levels among the identified classes. Because the distributions of IES-6 and PSI-4 item scores are skewed to the right, we used the non-parametric KW procedure to formally test our Hypothesis H_2_ and compare PTSD symptoms and COVID-19-related fears across the latent classes obtained in the previous step. Specifically, KW tests were conducted on the IES-6 and PSI-4 total scores of all individuals in the sample, and each sub-factor dimension across classes. When the tests produced significant differences (*p*-value < 0.1), post hoc analyses with Bonferroni-corrected multiple comparison tests were used to examine differences across the classes with Dunn’s test. To investigate differences between sexes (Hypothesis H_3_), KW tests and post hoc analyses were repeated on the sub-sample of male and female respondents separately. All the computations were conducted using the R statistical software, version 4.3.0.

## 3. Results

### 3.1. Descriptive Statistics

Table 1 reports the summary statistics of the variables in the dataset. Overall, the mean age was 45 years, 106 (33.33%) individuals were male and 212 (66.67%) were female. The mean BMI was 24.25 and a clear majority of individuals were nurses (44.65%) and non-frontline workers (88.36%). Moreover, the SW test indicated that the distributions of IES-6 and PSI-4 are not normally distributed (both *p*-values are less than 0.05), with a significant positive skew of 0.505 and 0.353 for IES-6 and PSI-4, respectively.

### 3.2. LCA Results

**Figure 2:**
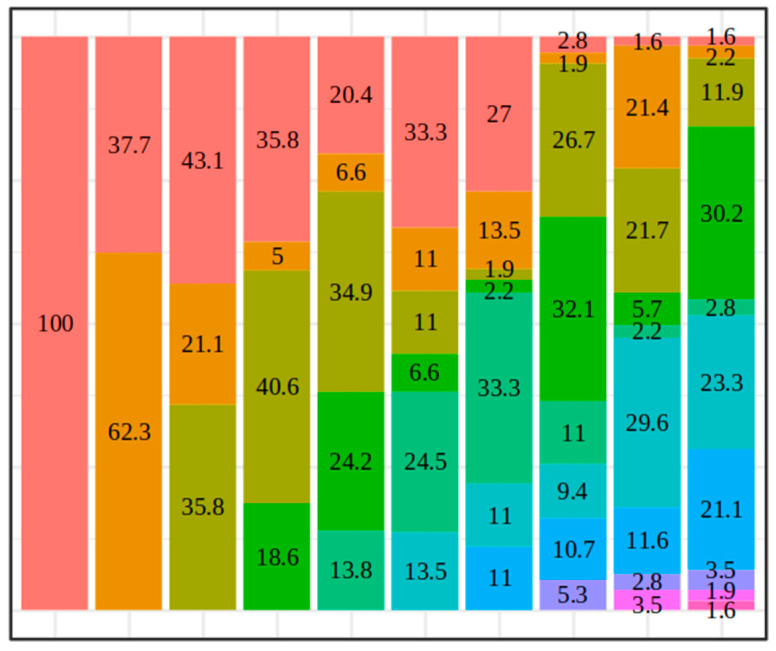
Latent class sizes (in percentage) based on the 11 COVID-19 symptoms from 1 to 10 classes.

Turning to the LCA results, Table 2 presents the results of the LCA for a varying number of latent classes from 1 up to 10, including the number of estimated parameters, the log-likelihood value, the entropy and several goodness-of-fit indices. In the last column labelled “Min. size” we also report the relative frequencies of the smallest latent class. According to the considered likelihood-based criteria, the BIC and the CAIC identified three distinct classes. The AWE produced a more parsimonious solution meanwhile the SABIC and AIC provided a four-class classification. However, it is well known that the AIC tends to overfit and select more complex models than the BIC. Moreover, in this second case, we obtained very small classes (5.0% of the total cases corresponding to 15 individuals) which could potentially lead to spurious results (Figure 2). Consistently with the BIC, to have an appropriate description of the phenomenon under study, we selected the three-class model. For additional insights on the obtained solutions from the LCA please refer to Figures S1–S3 in the Supplementary Materials.

**Figure 3:**
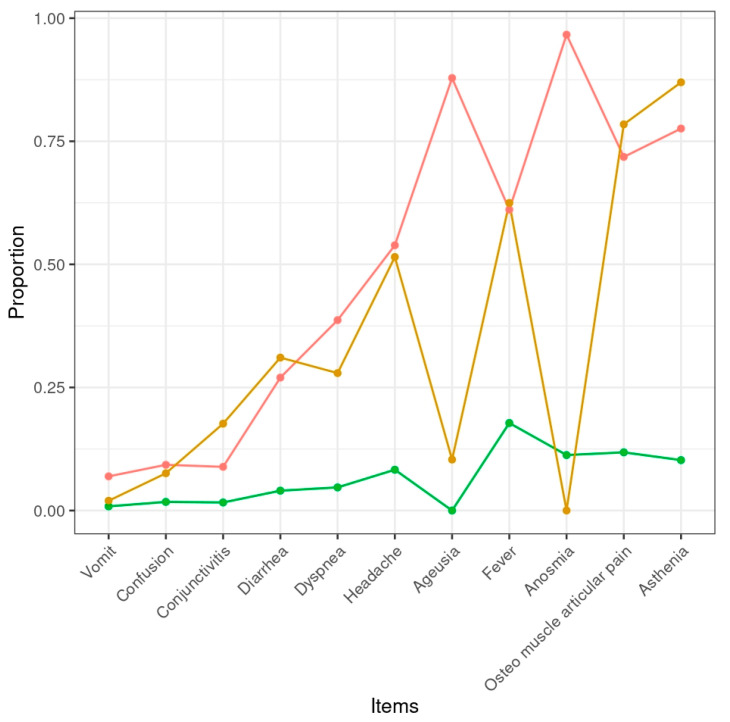
Characteristics of the latent classes based on the 11 COVID-19 symptoms for the three-class solution. Note: low, mild and high symptom classes are coloured in green, gold and red, respectively.

Once we chose the best model, we assigned each observation to one of the three classes based on their posterior class membership probability. Figure 3 illustrates the characteristics of the three subgroups based on the item responses to the 11 COVID-19 symptoms. As one can see, each class can be associated to different COVID-19 symptom severity: high symptoms class such as headache, ageusia and anosmia (*n* = 137, 43.1%; 95% CI, 36.3–49.9), moderate symptoms class without loss of taste and smell (*n* = 67, 21.1%; 95% CI, 14.0–28.2) and no/low symptoms class (*n* = 114, 35.8%; 95% CI, 29.5–42.2). Classes also differed with respect to gender composition: 28.9% and 15.4% of all the females belonged to the high and moderate symptoms classes, respectively. The resulting three-cluster solution supports our Hypothesis H_1_ which distinguishes distinct degrees of severity of COVID-19 symptomatology.

### 3.3. Results for KW Tests and Post Hoc Analyses

We now discuss the results of the KW tests and post hoc analyses conducted on the total scores of the IES-6 and PSI-4, and each of their sub-factors. Table 3 reports the mean and standard deviation of the scores in each latent class, along with the computed KW statistics and *p*-values. We respectively labelled the high, mild and low symptoms classes as C1, C2 and C3, meanwhile, the column named “post hoc” indicates whether significant differences (*p* < 0.1) between the mean rank of the latent classes exist. The KW test confirmed significant differences in the total IES-6 (KW = 11.087, *p* = 0.004) and PSI-4 (KW = 11.939, *p* = 0.003) scores for the entire sample, showing that more severe COVID-19 symptoms are accompanied by higher levels of PTSD and fears-related COVID-19. With the exception of “Many things make me think of COVID-19” and “Trying not to think about COVID-19” dimensions, all the other sub-factors were significantly different between the three classes. The post hoc analyses indicated that the first latent class (C1) scored significantly higher levels than the second and third ones (C2 and C3) across all dimensions of the IES-6 and PSI-4 questionnaires, supporting Hypothesis H_2_. However, the second and third classes were generally not significantly different.

Regarding the gender-related differences (Hypothesis H_3_), by looking at Table 3a,b, all female classes showed higher levels of PTSD than males, consistently with the composition of the latent groups. Specifically, the female sub-groups exhibited significant differences in both IES-6 (KW = 8.828, *p* = 0.012) and PSI-4 (KW = 5.741, *p* = 0.057) total scores, as well as in the dimensions “Felt nervous and alarmed”, “Negative emotions without realizing it” and “Difficulty concentrating”, confirming our Hypothesis H_3_. On the contrary, significant differences between the latent classes of the male groups were not present in general except for “Difficulty concentrating” (*p* = 0.045) and total PSI-4 scores (*p* = 0.060). Post hoc analyses suggested that the females in the first latent class experienced COVID-19-related anxiety and stress scores that were significantly higher than those of the second and third classes. Overall, these results reinforced the existence of different levels of PTSD and COVID-19-related fears among infected HCWs and provided further evidence in favour of Hypothesis H_3_.

## 4. Discussion

Although the pandemic represents a significant risk for all categories of workers, HCWs show greater vulnerability. During a pandemic, the number of patients is constantly increasing, limiting available healthcare resources, and increasing the level of discomfort of HCWs. Furthermore, all healthcare personnel have a greater risk of contracting COVID-19, due to constant direct exposure to hospital environments and possible COVID-19-positive patients. Indeed, this turns out to be a further stressor that can compromise the physical and mental health of HCWs [40]. A study by Greene and colleagues [41] conducted on a sample of frontline HCWs in 2021 revealed that approximately 54% of respondents exhibited clinically significant symptoms of anxiety, depression, and posttraumatic stress disorder. This percentage was higher in healthcare workers who had been affected by COVID infection. In this regard, the present study examined the relationship between the severity of COVID-19 symptoms and the risk of COVID-19-related fear and PTSD symptoms by dividing the sample into groups according to the level of severity of the symptomatic picture understood as the number of symptoms during COVID-19 infection. In addition, to investigate gender differences, we separated the sample by sex before conducting LCA. Our results revealed that more severe COVID-19 symptoms, and thus a higher number of symptoms, are associated with higher levels of PTSD and COVID-19 fear.

In fact, as highlighted by COR theory, individuals with an initial vulnerability can perceive greater difficulty in dealing with other challenging stimuli, experiencing a possible loss cycle of resources and thus undermining coping strategies [20,22]. In line with the COR theory principles, when the individual experiences a threat to their safety, understood as an attack on their health, this translates into a traumatic response [22]. Indeed, as shown by previous studies, contact with infected people or symptoms of COVID-19 infection increases the level of psychological consequences such as anxiety and PTSD [42,43]. Moreover, it has been seen how the uncertainty dictated by the symptomatic outcomes of the infection can reduce social interactions between individuals and increase the risk of depression and anxiety [44]. In particular, the population of HCWs who have been affected by COVID-19 are more likely to present symptoms of depression, anxiety, stress, and PTSD [10,45].

The results of our research showed that the group with a high number of physical symptoms presented more fears and PTSD symptoms than the other groups. This suggests to us, also in accordance with COR theory, that the vulnerability induced by the presence of a high number of physical symptoms from which HCWs suffer exposes them to a higher risk of fears and PTSD compared to those with a lower number of symptoms. As shown in the existing literature, the number of physical symptoms correlates with the number of psychological symptoms [46,47,48,49]. For example, the study by Keijsers and colleagues [49] showed that the presence of physical symptoms was correlated with high anxiety and depression scores and in line with our results, the literature confirms that a greater burden of physical symptoms was associated with higher IES scores [46].

Furthermore, the results of our study showed that females exhibited higher IES-6 and PSI-4 scores than males. The COVID-19 pandemic considered a high-impact traumatic event, has generated a higher risk of post-traumatic symptoms for women [36]. In line with the high scores on the PSI-4 scale, women report acute emotional responses more frequently than men [50], and experience intense fear, panic, depression and anxiety as well as intrusive thoughts and avoidance [51]. Regarding a possible neurobiological explanation, women with PTSD were found to have lower baseline cortisol levels than the control group. This association was not significant for males, and this has led to the hypothesis that women have a high sensitivity to variations in the hypothalamic–pituitary–adrenal axis and are consequently more exposed to the risk of developing PTSD [52]. Furthermore, consistent with the existing literature [53], the results of our research show that, among HCWs with physical symptoms, women have a higher prevalence of PTSD symptoms compared to men. All the studies mentioned confirming our results showed higher levels of PTSD than that of males. Our results suggest that exploring different levels of mental health can provide a more comprehensive understanding of the potential situations that may arise within the workplace context. In line with the study by Mazor and colleagues [46], in our study, we observed that subjects in the group with elevated symptoms exhibited higher levels of intrusion, hyperarousal, and avoidance compared to groups with moderate symptoms and those with mild/absent symptoms. Furthermore, the group with the poorest level of mental health also showed greater fear of contracting the virus, of being quarantined, of worsening pre-existing health conditions, and of transmitting the infection to their loved ones. This is consistent with COR Theory [20] and the concept of resource caravans. Stressful events prompt individuals to build so-called resource caravans [54]. This notion is based on the idea that resources do not exist in isolation but travel together to shield against potential resource loss. Consequently, experiencing a persistent condition of vulnerability dictated by the presence of severe physical symptoms can lead to a loss of resources that renders the individual vulnerable even from a psychological point of view.

In particular, women belonging to the group with severe symptoms exhibited higher scores of fears and PTSD, especially in the dimension of hyperarousal, experiencing greater negative emotions. These findings support studies that emphasize gender differences in traumatic responses [30,31,32] and existing literature regarding the increased risk women of experiencing distress and PTSD compared to men [29,36]. This allows us to understand the different degrees of mental health that are affected by the symptoms of COVID-19, differentiated by gender, and also allows us to extend this evidence to other phenomena that can jeopardize the health of workers.

In conclusion, this study has shed light on the relationship between COVID-19 symptomatology, focusing specifically on severity, and the risk of COVID-related fears and PTSD in a sample of HCWs. This is the population most at risk during epidemics. For this reason, the results of this study offer help in understanding the physical and psychological impact of a traumatic scenario such as COVID-19 for the creation of future prevention plans and emergency management.

### 4.1. Limitations and Future Research Direction

Despite the novelty of our results, this research has some limitations that should be addressed. First, a retrospective cross-sectional study was conducted and consequently, the observed relationship between COVID-19 symptoms and stress indicators has no causal interpretation. To address this relevant issue, future studies should consider longitudinal designs and compare results with our findings. Second, we used self-report measures to evaluate PTSD symptoms and COVID-19-related fear that may increase the risk of self-report common method bias, although there is still no unanimity on the magnitude of its effects [55]. Nevertheless, in line with the recommendations of Podskaoff et al., [56] we provided participants with information about the privacy of their responses, and the absence of wrong answers, and, moreover, we divided predictor and criterion variables sections in the survey questionnaires. The use of clinical tests or biological markers would increase the validity of our results. Third, we used a non-probabilistic sampling technique. We recruited the sample through a convenience sampling procedure, which may have undermined the generalization of our results. However, our sample was composed of a heterogeneous group of people, covering different age groups with a balanced gender distribution. Moreover, we can mention the recommendations offered by Wheeler and colleagues who assert this method of data acquisition has a good level of validity and reliability and is usually employed in organizational psychology [57]. Lastly, the heterogeneity of the sample determined by the multicentric study design could represent yet another potential source of bias. Indeed, results could have been affected by disparities in the geographic prevalence of the infection as well as different epidemiological and behavioural factors. Furthermore, the study does not consider potential confounding variables such as pre-existing mental health conditions, personal coping strategies, or support systems that might affect the psychological outcomes and should be addressed in future research. Finally, the results on gender are not representative of the Italian population, and there may also be cross-cultural differences in the perception of greater female distress found in the study.

### 4.2. Practical Implications

On a practical level, our results could be useful for designing adequate prevention and health promotion plans. Organizations should create and implement multi-level stress management practices, with particular attention to high-risk workers. From a managerial point of view and from a health prevention perspective, our results highlight the need to consider gradations of mental health and establish specific measures depending on the situation (e.g., employees might have different levels of stress and could be distinguished into healthy employees, employees at risk of mental problems, and employees with mental problems). Specific health prevention plans can be designed to intervene at different levels, i.e., primary prevention (before the development of symptoms), secondary prevention (to avoid long-term distress) and tertiary prevention (to treat the developed symptoms) [58]. In accordance with guidelines for trauma-exposed work environments, such as those from the National Institute for Health and Care Excellence (NICE), certain behavioral and psycho-social interventions can be beneficial in the early stages following a traumatic event. Initially, “active monitoring” is recommended, followed by interventions like memory restructuring, psychoeducation, and worksite crisis sessions, which have shown beneficial effects [59]. Hospital management should also provide targeted programs to train HCWs in dealing with potentially stressful events [60]. These programs can feature a variety of interventions, available both on-site and remotely. Remote resources might include telephone hotlines for immediate access to mental health support during acute distress [61] and online training packages with interactive modules covering topics such as cognitive–behavioral techniques, mindfulness strategies, assessment tools, and resilience-building exercises [62]. During the COVID-19 pandemic, different kinds of online/mobile resources designed to meet HCWs’ needs were developed. For example, Weiner et al., [63] developed a 7-sessions online program to teach HCWs coping strategies based on emotional regulation while Serrano-Ripoll et al., [64] tested the efficacy of a mobile app based on CBT, mindfulness and resources on lifestyle, and emotional regulation, work-related stress and burnout, which includes a personalized program based on users’ answers.

Moreover, organizations should pay particular attention to gender differences when designing interventions as women experience and react to traumatic experiences differently [65]. For example, women tend to use emotionally focused coping and avoidant coping more than men [66]. Since avoidance strategies are associated with higher PTSD symptoms and psychological distress, to improve coping strategies, female workers could be involved in targeted workshops aimed at reappraisal of trauma using active cognitive techniques (e.g., reframing) [67]. Especially for women, the lack of a supportive environment is one of the greatest predictors of trauma as they also seek and rely on social support more than men. The aim of organizations should be to design ad hoc interventions to promote workers’ networks of relationships [68]. This can be achieved using specific practices (e.g., small group discussions, digital communication channels) and through health-focused leadership (i.e., building a sense of shared self-efficacy and healthy relationships). Moreover, research suggests that interventions designed to develop the peer support system within teams, such as Trauma Risk Management (TRiM), can be effective in reducing stigma and improving workers’ ability to provide support [69,70]. Women also tend to experience higher levels of negative emotions such as posttraumatic symptoms (e.g., hyperarousal), intense fear, panic, depression and anxiety [71]. Greater awareness and management of emotions could be effective in reducing psychological distress. In this regard, awareness of physical and psychological symptoms can be promoted through psychoeducation interventions. Effective programs can include specific training and counseling services such as interpersonal counselling, preventive counselling and debriefing sessions to promote resilience [72]. Organizations should design, implement and monitor tailored policies, provide training about the physical and psychological manifestations of trauma and establish a process for screening high-risk employees [73]. Indeed, to structure effective interventions that match the needs of workers, it would be advisable to plan an initial evaluation phase with ad hoc investigations aimed at assessing physical and psychological health. For example, a recent systematic review highlighted the importance of workplace health promotion programs aimed at addressing both mental and physical health (e.g., promoting healthy habits and giving access to mental health services) [74].

## 5. Conclusions

The results of our study indicate that a critical symptom picture can have a significant impact on worker well-being, especially during traumatic scenarios such as the COVID-19 pandemic and for at-risk populations such as HCWs. In this regard, these findings shed light on the possible outcomes of high-impact events, i.e., what the possible effects of physical symptoms could be and how these can lead to an increase in psychological symptoms such as fears and PTSD. Gender differences in the level of COVID-19-related fears and post-traumatic stress disorder were also highlighted.

## Figures and Tables

**Table 1:** Descriptive statistics of the sample.

Variables	Total (*n* = 318)
**Gender**	
Male	106 (33.33%)
Female	212 (66.67%)
**Age, years**	45.07 (11.93)
**BMI, kg/m^2^**	24.25 (4.787)
**Job**	
Nurse	142 (44.65%)
Healthcare Assistant	68 (21.38%)
Doctor	34 (10.69%)
Resident Doctor	37 (11.64%)
Other	37 (11.64%)
**Frontline**	
No	281 (88.36%)
Yes	37 (11.64%)
**Total IES-6**	14.97 (6.205)
**Total PSI-4**	11.47 (3.887)
**Vomit**	
No	306 (96.23%)
Yes	12 (3.77%)
**Confusion**	
No	298 (93.71%)
Yes	20 (6.29%)
**Conjunctivitis**	
No	292 (91.82%)
Yes	26 (8.18%)
**Diarrhea**	
No	255 (80.19%)
Yes	63 (19.81%)
**Dyspnea**	
No	240 (75.47%)
Yes	78 (24.53%)
**Headache**	
No	199 (62.58%)
Yes	119 (37.42%)
**Ageusia**	
No	188 (59.12%)
Yes	130 (40.88%)
**Fever**	
No	171 (53.77%)
Yes	147 (46.23%)
**Anosmia**	
No	170 (53.46%)
Yes	148 (46.54%)
**Osteo muscle articular pain**	
No	152 (47.80%)
Yes	166 (52.20%)
**A** **sthenia**	
No	140 (44.03%)
Yes	178 (55.97%)

Note: For continuous variables we report the mean and standard deviation (in parenthesis) while for categorical ones we report the absolute frequency and percentage frequency (in parenthesis).

**Table 2:** Fit statistics for a varying number of latent classes from 1 to 10.

Classes	Npar	−2LL	AIC	BIC	AWE	CAIC	SABIC	Entropy	Min. Size
1	11	3708.04	3730.04	3771.42	3856.14	3782.42	3736.53	-	1.00
2	23	3283.90	3329.90	3416.43	**3607.59**	3439.43	3343.48	0.84	0.39
3	35	3171.60	3241.60	**3373.27**	3669.95	**3408.27**	3262.26	0.88	0.21
4	47	3133.35	**3227.35**	3404.16	3806.11	3451.16	**3255.09**	0.85	0.06
5	59	3112.48	3230.48	3452.44	3959.62	3511.44	3265.31	0.92	0.05
6	71	3087.94	3229.94	3497.05	4109.44	3568.05	3271.85	0.88	0.07
7	83	3072.48	3238.48	3550.73	4268.32	3633.73	3287.47	0.90	0.01
8	95	3061.02	3251.02	3608.42	4431.19	3703.42	3307.10	0.88	0.03
9	107	3042.02	3256.02	3658.56	4586.53	3765.56	3319.18	0.93	0.02
10	119	3034.72	3272.72	3720.41	4753.54	3839.41	3342.96	0.84	0.01

Note: Model selection criteria comparing the quality of different solutions (the lower the better). Bold text indicates the best solution for each criterion. Npar = number of estimated parameters; −2LL = minus twice the log-likelihood; AIC = Akaike information criterion; BIC = Bayesian information criterion; SABIC = sample-size adjusted BIC; AWE = approximate weight of evidence criterion; CAIC = consistent Akaike information criterion. The column “Min. Size” reports the relative frequencies of the smallest latent class.

**Table 3:** Results for Kruskal–Wallis test on fears of COVID-19, COVID-19-related PTSD, and each sub-factor dimension. (**a**) males; (**b**) females.

Variables	C1		C2		C3		KW	*p*	Post Hoc
	Mean	SD	Mean	SD	Mean	SD			
**Total IES-6**	16.197	6.357	14.522	5.434	13.746	6.218	11.087	0.004 **	C1 > C3
Many things make me think of COVID-19	3.161	1.307	3.164	1.214	3.009	1.32	0.968	0.616	
Thinking about COVID-19 even without wanting to	2.547	1.419	2.358	1.264	2.158	1.301	4.813	0.090 ^+^	C1 > C3
Felt nervous and alarmed	2.599	1.309	2.284	1.229	2.228	1.175	6.005	0.050 ^+^	C1 > C3
Trying not to think about COVID-19	2.737	1.467	2.269	1.136	2.447	1.364	4.658	0.097 ^+^	
Negative emotions without realizing it	2.642	1.413	2.164	1.274	2.07	1.274	11.827	0.003 **	C1 > C2; C1 > C3
Difficulty concentrating	2.511	1.446	2.284	1.312	1.833	1.336	18.309	0.000 ***	C1 > C3; C2 > C1
**Total PSI-4**	12.226	3.86	11.463	3.767	10.57	3.828	11.939	0.003 **	C1 > C3
Contracting COVID-19	2.883	1.415	2.507	1.364	2.404	1.387	8.184	0.017 *	C1 > C3
Being quarantined	2.964	1.507	2.672	1.408	2.456	1.47	7.529	0.023 *	C1 > C3
Aggravating existing diseases	2.044	1.46	2.104	1.383	1.702	1.24	5.647	0.059 ^+^	C2 > C3
Infecting family members	4.336	1.038	4.179	1.23	4.009	1.244	4.742	0.093 ^+^	C1 > C3
**(a)**									
**Variables**	**C1**		**C2**		**C3**		**KW**	***p***	**Post Hoc**
	**Mean**	**SD**	**Mean**	**SD**	**Mean**	**SD**			
**Total IES-6**	14.022	6.276	13.611	4.779	12.116	4.924	2.346	0.309	
Many things make me think of COVID-19	2.822	1.386	2.944	1.259	2.86	1.265	0.242	0.886	
Thinking about COVID-19 even without wanting to	2.267	1.421	2	0.907	1.907	1.211	1.243	0.537	
Felt nervous and alarmed	2.267	1.176	2.5	1.383	1.837	0.924	4.384	0.112	
Trying not to think about COVID-19	2.533	1.517	2.056	0.938	2.279	1.26	0.888	0.641	
Negative emotions without realizing it	2.089	1.258	2.167	1.249	1.651	0.997	3.919	0.141	
Difficulty concentrating	2.044	1.224	1.944	1.056	1.581	1.118	6.223	0.045 *	C1 > C3
**Total PSI-4**	11.156	3.766	10.333	3.236	9.419	2.978	5.637	0.060 ^+^	C1 > C3
Contracting COVID-19	2.644	1.479	2	1.138	2.093	1.25	3.998	0.135	
Being quarantined	2.644	1.583	2.444	1.042	2.047	1.234	3.815	0.148	
Aggravating existing diseases	1.778	1.396	1.833	1.339	1.395	1.003	3.207	0.201	
Infecting family members	4.089	1.184	4.056	1.211	3.884	1.258	0.74	0.691	
**(b)**									
**Variables**	**C1**		**C2**		**C3**		**KW**	***p***	**Post Hoc**
	**Mean**	**SD**	**Mean**	**SD**	**Mean**	**SD**			
**Total IES-6**	17.261	6.153	14.857	5.664	14.732	6.727	8.828	0.012 *	C1 > C3; C1 > C2
Many things make me think of COVID-19	3.326	1.241	3.245	1.199	3.099	1.354	1.088	0.580	
Thinking about COVID-19 even without wanting to	2.685	1.406	2.49	1.356	2.31	1.337	3.01	0.222	
Felt nervous and alarmed	2.761	1.345	2.204	1.172	2.465	1.252	6.21	0.045 *	C1 > C2
Trying not to think about COVID-19	2.837	1.439	2.347	1.2	2.549	1.422	3.897	0.143	
Negative emotions without realizing it	2.913	1.412	2.163	1.297	2.324	1.36	11.555	0.003 **	C1 > C3; C1 > C2
Difficulty concentrating	2.739	1.496	2.408	1.383	1.986	1.439	11.518	0.003 **	C1 > C3
**Total PSI-4**	12.75	3.816	11.878	3.892	11.268	4.126	5.741	0.057 ^+^	C1 > C3
Contracting COVID-19	3	1.375	2.694	1.402	2.592	1.44	3.944	0.139	
Being quarantined	3.12	1.451	2.755	1.521	2.704	1.553	3.796	0.150	
Aggravating existing diseases	2.174	1.48	2.204	1.399	1.887	1.337	2.157	0.340	
Infecting family members	4.457	0.942	4.224	1.246	4.085	1.239	3.82	0.148	

Note: C1, C2 and C3 denote the latent classes characterized as high, moderate and low symptoms classes, respectively. SD = standard deviation; KW = Kruskal–Wallis statistic; *p* = *p*-value. The Post hoc column reports significant differences in Bonferroni-corrected multiple comparisons between latent classes (*p* < 0.1) using Dunn’s test. ^+^ *p* < 0.10; * *p* < 0.05; ** *p* < 0.01; *** *p* < 0.001.

## Data Availability

Data are available from the authors upon reasonable request.

## Supplementary Materials

The following supporting information can be downloaded at: https://www.mdpi.com/article/10.3390/healthcare12141403/s1, Figure S1: diagnostic criteria (left) and log-likelihood (right) values for the fitted models over 1, …, 10 latent classes; Figure S2: Characteristics of the latent classes based on the 11 COVID-19 symptoms with a varying number of latent classes from 1 to 6; Figure S3: Average latent posterior probability matrices for a varying number of latent classes from 1 to 10. The administered questionnaire to the HCWs is made available in both Italian and English.

## References

[1] Li Q., Guan X., Wu P., Wang X., Zhou L., Tong Y., Ren R., Leung K.S.M., Lau E.H.Y., Wong J.Y. (2020). Early Transmission Dynamics in Wuhan, China, of Novel Coronavirus–Infected Pneumonia. N. Engl. J. Med..

[2] Guan W., Ni Z., Hu Y., Liang W., Ou C., He J., Liu L., Shan H., Lei C., Hui D.S.C. (2020). Clinical Characteristics of Coronavirus Disease 2019 in China. N. Engl. J. Med..

[3] Wang D., Hu B., Hu C., Zhu F., Liu X., Zhang J., Wang B., Xiang H., Cheng Z., Xiong Y. (2020). Clinical Characteristics of 138 Hospitalized Patients with 2019 Novel Coronavirus–Infected Pneumonia in Wuhan, China. JAMA.

[4] Filindassi V., Pedrini C., Sabadini C., Duradoni M., Guazzini A. (2022). Impact of COVID-19 First Wave on Psychological and Psychosocial Dimensions: A Systematic Review. COVID.

[5] Ahorsu D.K., Lin C.-Y., Imani V., Saffari M., Griffiths M.D., Pakpour A.H. (2020). The Fear of COVID-19 Scale: Development and Initial Validation. Int. J. Ment. Health Addict..

[6] Yuen K.F., Wang X., Ma F., Li K.X. (2020). The Psychological Causes of Panic Buying Following a Health Crisis. Int. J. Environ. Res. Public Health.

[7] Aydin C. (2017). How to Forget the Unforgettable? On Collective Trauma, Cultural Identity, and Mnemotechnologies. Identity.

[8] Bridgland V.M.E., Moeck E.K., Green D.M., Swain T.L., Nayda D.M., Matson L.A., Hutchison N.P., Takarangi M.K.T. (2021). Why the COVID-19 Pandemic Is a Traumatic Stressor. PLoS ONE.

[9] Sanchez-Gomez M., Giorgi G., Finstad G.L., Urbini F., Foti G., Mucci N., Zaffina S., León-Perez J.M. (2021). COVID-19 Pandemic as a Traumatic Event and Its Associations with Fear and Mental Health: A Cognitive-Activation Approach. Int. J. Environ. Res. Public Health.

[10] Rossi R., Socci V., Pacitti F., Di Lorenzo G., Di Marco A., Siracusano A., Rossi A. (2020). Mental Health Outcomes among Frontline and Second-Line Health Care Workers during the Coronavirus Disease 2019 (COVID-19) Pandemic in Italy. JAMA Netw. Open.

[11] Di Tella M., Romeo A., Benfante A., Castelli L. (2020). Mental Health of Healthcare Workers during the COVID-19 Pandemic in Italy. Eval. Clin. Pract..

[12] Zakeri M.A., Hossini Rafsanjanipoor S.M., Sedri N., Kahnooji M., Sanji Rafsanjani M., Zakeri M., Zakeri Bazmandeh A., Talebi A., Dehghan M. (2021). Psychosocial Status during the Prevalence of COVID-19 Disease: The Comparison between Healthcare Workers and General Population. Curr. Psychol..

[13] Ferrat E., Mirat W., Boutin E., Maroto E., Brossier S., Hoonakker J.-D., Audureau E., Phan T.-T., Bastuji-Garin S. (2024). COVID-19 Profiles in General Practice: A Latent Class Analysis. BMJ Open.

[14] Moreira R.D.S. (2021). Análises de Classes Latentes Dos Sintomas Relacionados à COVID-19 No Brasil: Resultados Da PNAD-COVID19. Cad. De Saúde Pública.

[15] Moradi-Asl E., Adham D., Ghobadi H., Abbasi-Ghahramanloo A. (2021). Clustering of COVID-19 Symptoms among Iranian Patients: The Role of Preexisting Comorbidity on Latent Class Membership. Asia Pac. J. Public Health.

[16] Malik S., Ullah I., Irfan M., Ahorsu D.K., Lin C.-Y., Pakpour A.H., Griffiths M.D., Rehman I.U., Minhas R. (2021). Fear of COVID-19 and Workplace Phobia among Pakistani Doctors: A Survey Study. BMC Public Health.

[17] Sekowski M., Gambin M., Hansen K., Holas P., Hyniewska S., Wyszomirska J., Pluta A., Sobańska M., Łojek E. (2021). Risk of Developing Post-Traumatic Stress Disorder in Severe COVID-19 Survivors, Their Families and Frontline Healthcare Workers: What Should Mental Health Specialists Prepare For?. Front. Psychiatry.

[18] Mak I.W.C., Chu C.M., Pan P.C., Yiu M.G.C., Ho S.C., Chan V.L. (2010). Risk Factors for Chronic Post-Traumatic Stress Disorder (PTSD) in SARS Survivors. Gen. Hosp. Psychiatry.

[19] Mohammadian Khonsari N., Shafiee G., Zandifar A., Mohammad Poornami S., Ejtahed H.-S., Asayesh H., Qorbani M. (2021). Comparison of Psychological Symptoms between Infected and Non-Infected COVID-19 Health Care Workers. BMC Psychiatry.

[20] Hobfoll S.E. (1989). Conservation of Resources: A New Attempt at Conceptualizing Stress. Am. Psychol..

[21] Hobfoll S.E. (2001). The Influence of Culture, Community, and the Nested-Self in the Stress Process: Advancing Conservation of Resources Theory. Appl. Psychol..

[22] Hobfoll S.E., Halbesleben J., Neveu J.-P., Westman M. (2018). Conservation of Resources in the Organizational Context: The Reality of Resources and Their Consequences. Annu. Rev. Organ. Psychol. Organ. Behav..

[23] Boone A., Vander Elst T., Vandenbroeck S., Godderis L. (2022). Burnout Profiles among Young Researchers: A Latent Profile Analysis. Front. Psychol..

[24] Leiter M.P., Maslach C. (2016). Latent Burnout Profiles: A New Approach to Understanding the Burnout Experience. Burn. Res..

[25] Frounfelker R.L., Li Z.Y., Santavicca T., Miconi D., Rousseau C. (2022). Latent Class Analysis of COVID-19 Experiences, Social Distancing, and Mental Health. Am. J. Orthopsychiatry.

[26] Yu Y., Lau J.T.F., Lau M.M.C. (2023). Development and Validation of the Conservation of Resources Scale for COVID-19 in the Chinese Adult General Population. Curr. Psychol..

[27] Lazarsfeld P.F. (1950). The Logical and Mathematical Foundation of Latent Structure Analysis. Studies in Social Psychology in World War II Vol. IV: Measurement and Prediction.

[28] Lazarus R.S., Folkman S. (1984). Stress, Appraisal, and Coping.

[29] Fryers T., Brugha T., Morgan Z., Smith J., Hill T., Carta M., Lehtinen V., Kovess V. (2004). Prevalence of Psychiatric Disorder in Europe: The Potential and Reality of Meta-Analysis. Soc. Psychiatry Psychiatr. Epidemiol..

[30] Ehlers A., Clark D.M. (2000). A Cognitive Model of Posttraumatic Stress Disorder. Behav. Res. Ther..

[31] Foa E.B., Riggs D.S., Gershuny B.S. (1995). Arousal, Numbing, and Intrusion: Symptom Structure of PTSD Following Assault. Am. J. Psychiatry.

[32] Horowitz M.J. (1986). Stress-Response Syndromes: A Review of Posttraumatic and Adjustment Disorders. Psychiatr. Serv..

[33] Mak A.S., Blewitt K., Heaven P.C.L. (2004). Gender and Personality Influences in Adolescent Threat and Challenge Appraisals and Depressive Symptoms. Personal. Individ. Differ..

[34] Ptacek J.T., Smith R.E., Zanas J. (1992). Gender, Appraisal, and Coping: A Longitudinal Analysis. J. Personal..

[35] Eisler R.M., Skidmore J.R. (1987). Masculine Gender Role Stress: Scale Development and Component Factors in the Appraisal of Stressful Situations. Behav. Modif..

[36] Ahuja P., Syal G., Kaur A. (2021). Psychological Stress: Repercussions of COVID-19 on Gender. J. Public Aff..

[37] Dickerson S.S., Kemeny M.E. (2004). Acute Stressors and Cortisol Responses: A Theoretical Integration and Synthesis of Laboratory Research. Psychol. Bull..

[38] McClure E.B., Monk C.S., Nelson E.E., Zarahn E., Leibenluft E., Bilder R.M., Charney D.S., Ernst M., Pine D.S. (2004). A Developmental Examination of Gender Differences in Brain Engagement during Evaluation of Threat. Biol. Psychiatry.

[39] Giorgi G., Fiz Perez F., Castiello D’Antonio A., Mucci N., Ferrero C., Cupelli V., Arcangeli G. (2015). Psychometric Properties of the Impact of Event Scale-6 in a Sample of Victims of Bank Robbery. Psychol. Res. Behav. Manag..

[40] Shiao J.S.-C., Koh D., Lo L.-H., Lim M.-K., Guo Y.L. (2007). Factors Predicting Nurses’ Consideration of Leaving Their Job during the Sars Outbreak. Nurs. Ethics.

[41] Greene T., Harju-Seppänen J., Adeniji M., Steel C., Grey N., Brewin C.R., Bloomfield M.A., Billings J. (2021). Predictors and Rates of PTSD, Depression and Anxiety in UK Frontline Health and Social Care Workers during COVID-19. Eur. J. Psychotraumatology.

[42] Cansel N., Ucuz İ., Arslan A.K., Kayhan Tetik B., Colak C., Melez Ş.N.İ., Şule Gümüstakım R., Ceylan S., Zeren Öztürk G., Kılıç Öztürk Y. (2021). Prevalence and Predictors of Psychological Response during Immediate COVID-19 Pandemic. Int. J. Clin. Pract..

[43] Bailey E.K., Steward K.A., VandenBussche Jantz A.B., Kamper J.E., Mahoney E.J., Duchnick J.J. (2021). Neuropsychology of COVID-19: Anticipated Cognitive and Mental Health Outcomes. Neuropsychology.

[44] Cao W., Fang Z., Hou G., Han M., Xu X., Dong J., Zheng J. (2020). The Psychological Impact of the COVID-19 Epidemic on College Students in China. Psychiatry Res..

[45] Chew N.W.S., Lee G.K.H., Tan B.Y.Q., Jing M., Goh Y., Ngiam N.J.H., Yeo L.L.L., Ahmad A., Ahmed Khan F., Napolean Shanmugam G. (2020). A Multinational, Multicentre Study on the Psychological Outcomes and Associated Physical Symptoms amongst Healthcare Workers during COVID-19 Outbreak. Brain Behav. Immun..

[46] Mazor M., Paul S.M., Chesney M.A., Chen L., Smoot B., Topp K., Conley Y.P., Levine J.D., Miaskowski C. (2019). Perceived Stress Is Associated with a Higher Symptom Burden in Cancer Survivors. Cancer.

[47] Tracy M.F., Hagstrom S., Mathiason M., Wente S., Lindquist R. (2024). Emotional, Mental Health and Physical Symptom Experience of Patients Hospitalized with COVID-19 up to 3 Months Post-hospitalization: A Longitudinal Study. J. Clin. Nurs..

[48] Kroenke K., Jackson J.L., Chamberlin J. (1997). Depressive and Anxiety Disorders in Patients Presenting with Physical Complaints. Am. J. Med..

[49] Keijsers K., Broeders M., Baptista Lopes V., Klinkert A., Van Baar J., Nahar-van Venrooij L., Kerckhoffs A. (2022). Memory Impairment and Concentration Problems in COVID-19 Survivors 8 Weeks after non-ICU Hospitalization: A Retrospective Cohort Study. J. Med. Virol..

[50] Brunet A., Weiss D.S., Metzler T.J., Best S.R., Neylan T.C., Rogers C., Fagan J., Marmar C.R. (2001). The Peritraumatic Distress Inventory: A Proposed Measure of PTSD Criterion A2. Am. J. Psychiatry.

[51] Freedman S.A., Gluck N., Tuval-Mashiach R., Brandes D., Peri T., Shalev A.Y. (2002). Gender Differences in Responses to Traumatic Events: A Prospective Study. J. Trauma. Stress.

[52] Irish L.A., Fischer B., Fallon W., Spoonster E., Sledjeski E.M., Delahanty D.L. (2011). Gender Differences in PTSD Symptoms: An Exploration of Peritraumatic Mechanisms. J. Anxiety Disord..

[53] Benfante A., Di Tella M., Romeo A., Castelli L. (2020). Traumatic Stress in Healthcare Workers During COVID-19 Pandemic: A Review of the Immediate Impact. Front. Psychol..

[54] Heath N.M., Hall B.J., Russ E.U., Canetti D., Hobfoll S.E. (2012). Reciprocal Relationships between Resource Loss and Psychological Distress Following Exposure to Political Violence: An Empirical Investigation of COR Theory’s Loss Spirals. Anxiety Stress. Coping.

[55] Spector P.E. (2006). Method Variance in Organizational Research: Truth or Urban Legend?. Organ. Res. Methods.

[56] Podsakoff P.M., MacKenzie S.B., Podsakoff N.P. (2012). Sources of Method Bias in Social Science Research and Recommendations on How to Control It. Annu. Rev. Psychol..

[57] Wheeler A.R., Shanine K.K., Leon M.R., Whitman M.V. (2014). Student-Recruited Samples in Organizational Research: A Review, Analysis, and Guidelines for Future Research. J. Occup. Organ. Psychol..

[58] Hollifield M., Gory A., Siedjak J., Nguyen L., Holmgreen L., Hobfoll S.E. (2016). The Benefit of Conserving and Gaining Resources after Trauma: A Systematic Review. J. Clin. Med..

[59] Brooks S.K., Rubin G.J., Greenberg N. (2019). Traumatic Stress within Disaster-Exposed Occupations: Overview of the Literature and Suggestions for the Management of Traumatic Stress in the Workplace. Br. Med. Bull..

[60] d’Ettorre G., Pellicani V., Ceccarelli G. (2020). Post-Traumatic Stress Disorder Symptoms in Healthcare Workers: A Ten-Year Systematic Review: Post-Traumatic Stress Disorder Symptoms in Healthcare Workers. Acta Biomed..

[61] Gray M., Monti K., Katz C., Klipstein K., Lim S. (2021). A “Mental Health PPE” Model of Proactive Mental Health Support for Frontline Health Care Workers during the COVID-19 Pandemic. Psychiatry Res..

[62] David E., DePierro J.M., Marin D.B., Sharma V., Charney D.S., Katz C.L. (2022). COVID-19 Pandemic Support Programs for Healthcare Workers and Implications for Occupational Mental Health: A Narrative Review. Psychiatr. Q..

[63] Weiner L., Berna F., Nourry N., Severac F., Vidailhet P., Mengin A.C. (2020). Efficacy of an Online Cognitive Behavioral Therapy Program Developed for Healthcare Workers during the COVID-19 Pandemic: The REduction of STress (REST) Study Protocol for a Randomized Controlled Trial. Trials.

[64] Serrano-Ripoll M.J., Meneses-Echavez J.F., Ricci-Cabello I., Fraile-Navarro D., Fiol-deRoque M.A., Pastor-Moreno G., Castro A., Ruiz-Pérez I., Zamanillo Campos R., Gonçalves-Bradley D.C. (2020). Impact of Viral Epidemic Outbreaks on Mental Health of Healthcare Workers: A Rapid Systematic Review and Meta-Analysis. J. Affect. Disord..

[65] Wade D., Varker T., Kartal D., Hetrick S., O’Donnell M., Forbes D. (2016). Gender Difference in Outcomes Following Trauma-Focused Interventions for Posttraumatic Stress Disorder: Systematic Review and Meta-Analysis. Psychol. Trauma Theory Res. Pract. Policy.

[66] Békés V., Beaulieu-Prévost D., Guay S., Belleville G., Marchand A. (2016). Women with PTSD Benefit More from Psychotherapy than Men. Psychol. Trauma Theory Res. Pract. Policy.

[67] Finstad G.L., Giorgi G., Lulli L.G., Pandolfi C., Foti G., León-Perez J.M., Cantero-Sánchez F.J., Mucci N. (2021). Resilience, Coping Strategies and Posttraumatic Growth in the Workplace Following COVID-19: A Narrative Review on the Positive Aspects of Trauma. Int. J. Environ. Res. Public Health.

[68] Wang Y., Chung M.C., Wang N., Yu X., Kenardy J. (2021). Social Support and Posttraumatic Stress Disorder: A Meta-Analysis of Longitudinal Studies. Clin. Psychol. Rev..

[69] Greenberg N., Langston V., Jones N. (2008). Trauma Risk Management (TRiM) in the UK Armed Forces. BMJ Mil. Health.

[70] Whybrow D., Jones N., Greenberg N. (2015). Promoting Organizational Well-Being: A Comprehensive Review of Trauma Risk Management: Table 1. Occup. Med..

[71] Kolakowsky-Hayner S.A., Goldin Y., Kingsley K., Alzueta E., Arango-Lasprilla J.C., Perrin P.B., Baker F.C., Ramos-Usuga D., Constantinidou F. (2021). Psychosocial Impacts of the COVID-19 Quarantine: A Study of Gender Differences in 59 Countries. Medicina.

[72] Kalaitzaki A., Rovithis M. (2021). Secondary Traumatic Stress and Vicarious Posttraumatic Growth in Healthcare Workers during the First COVID-19 Lockdown in Greece: The Role of Resilience and Coping Strategies. Psychiatriki.

[73] McFarlane A.C., Bryant R.A. (2007). Post-Traumatic Stress Disorder in Occupational Settings: Anticipating and Managing the Risk. Occup. Med..

[74] Joyce S., Modini M., Christensen H., Mykletun A., Bryant R., Mitchell P.B., Harvey S.B. (2016). Workplace Interventions for Common Mental Disorders: A Systematic Meta-Review. Psychol. Med..

