# Supplementary material for: COVID-19 Symptoms and Mental Health Outcomes among Italian Healthcare Workers: A Latent Class Analysis
Source: Healthcare (Basel). 2024 Jul 15;12(14):1403. doi: 10.3390/healthcare12141403 (PMC11275353; doi:10.3390/healthcare12141403)
Supplement: Supplementary file 1 [file healthcare-12-01403-s001.zip › Supplementary Materials figures.pdf]

Supplementary Materials for "COVID-19 symptoms and mental health outcomes among Italian healthcare workers: a latent class analysis"

Corresponding author: Giulia Foti, Giulia.Foti@unier.it

This Word file includes:

- Figure S1;
- Figure S2;
- Figure S3.

Additional figures for the analysis of HCWs

Figure S1: diagnostic criteria (left) and log-likelihood (right) values for the fitted models over 1, ..., 10 latent classes.

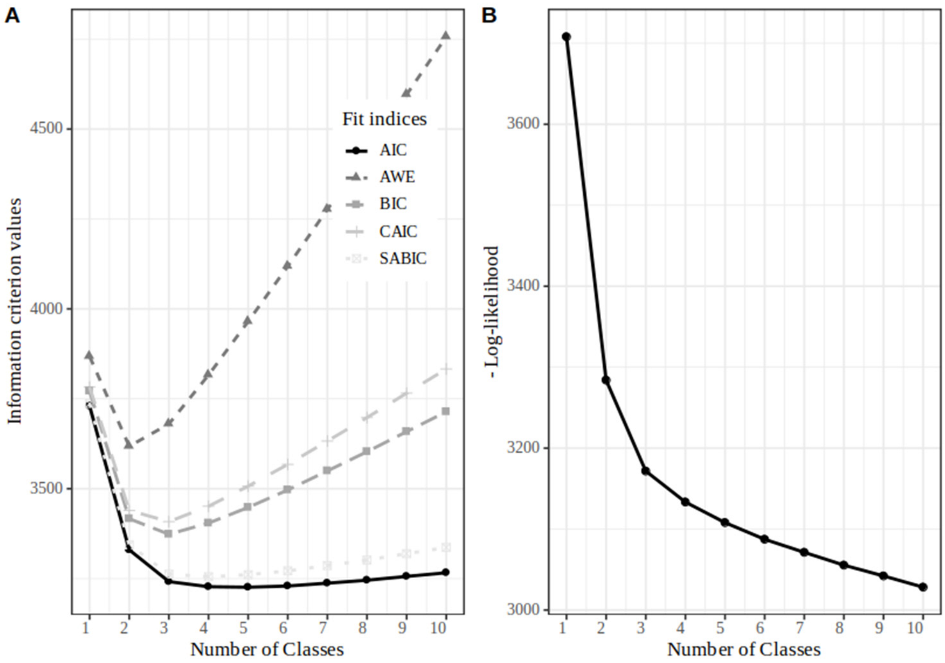

Figure S2: Characteristics of the latent classes based on the 11 COVID-19 symptoms with a varying number of latent classes from 1 to 6.

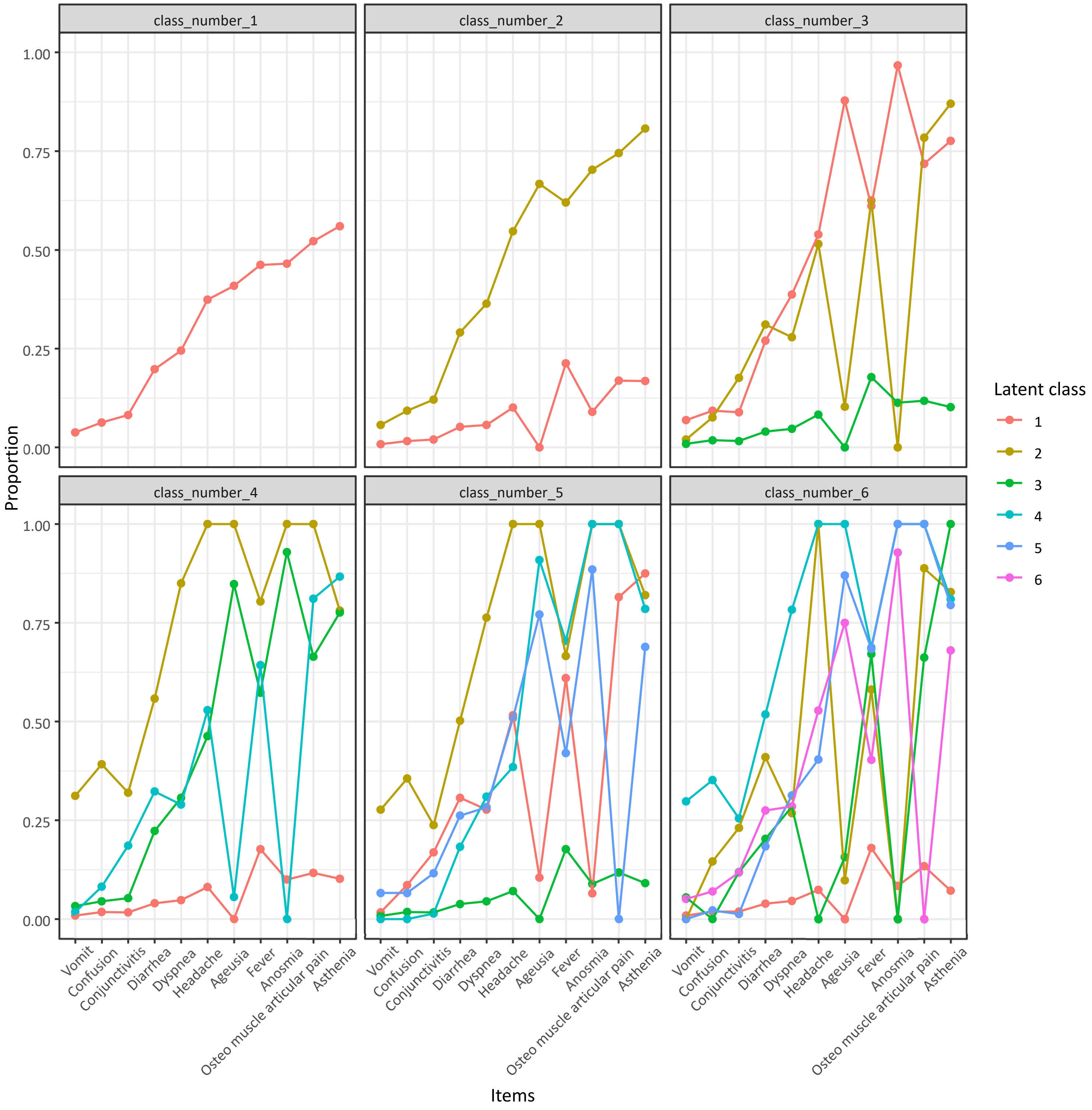

Figure S3: Average latent posterior probability matrices for a varying number of latent classes from 1 to 10.

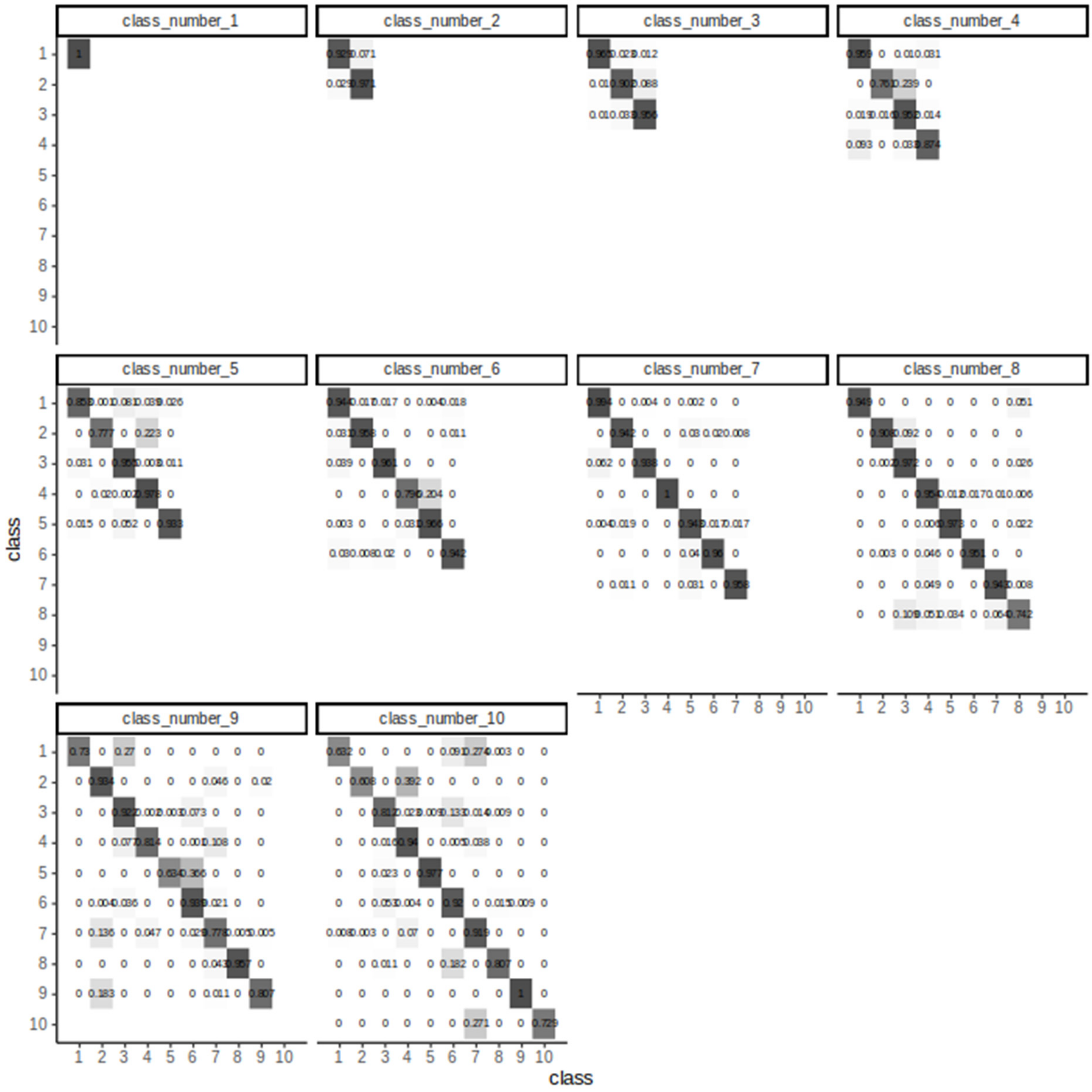

Note: The diagonals represent the average probability of a unit being assigned to a class given his/her scores on the indicator variables used to create the classes. Higher diagonal values (i.e., closer to 1, highlighted in dark gray) are desirable. Off-diagonal elements contain probabilities of cases that belong in one class being assigned to another class in the current solution. Lower values off the diagonal (i.e., closer to 0, highlighted in light gray) are desirable.
