# Supplementary material for: COVID-19 Symptoms and Mental Health Outcomes among Italian Healthcare Workers: A Latent Class Analysis
Source: Healthcare (Basel). 2024 Jul 15;12(14):1403. doi: 10.3390/healthcare12141403 (PMC11275353; doi:10.3390/healthcare12141403)
Supplement: Supplementary file 1 [file healthcare-12-01403-s001.zip › Supplementary Materials survey english v.pdf]

POST-CoViD-19 RE-ENTRY

PERSONAL AND WORK FORM			
SURNAME		NAME	
REGISTRATION NUMBER		GENDER	
MOBILE PHONE		EMAIL	
ROLE		DEPARTMENT	

R T - P C R S A R S - C o V - 2		
	DATE	RESULT
RT-PCR		
RT-PCR		
RT-PCR		
RT-PCR		

ACTIVE SURVEILLANCE	
RATED	
RISK INDEX MDL (if classified)	
NON-WORK CONTACTS	

COVID-19 DEVELOPMENT			
HOSPITALIZATION			
FEVER		ANOSMIA	
COUGH		AGEUSIA	
DYSPNEA		CONFUSION	
ASTENIA		VOMIT	
HEADACHE		DIARRHOEA	
CONJUNCTIVITIS		ASYMPTOMATIC	
OSTEO-MUSCULO-ARTICULAR PAIN		OTHER	
POSITIVITY DURATION			
NOTES / THERAPY			

P O S T C o V i D - 1 9			
ASYMPTOMATIC		ANOSMIA	
COUGH		AGEUSIA	
DYSPNEA		CONFUSION	
ASTENIA		CONJUNCTIVITI S	
HEADACHE		OSTEO-MUSCULO-ARTICULAR PAIN	

POST-CoViD-19 RE-ENTRY

NOTES / THERAPY	

POST-CoViD-19 RE-ENTRY

REMOTE PATHOLOGICAL HISTORY		
**CARDIOVASCULAR D.**		
**PNEUMOLOGICAL D.**		
**NEUROLOGICAL D.**		
**PSYCHIATRIC D.**		
**DIABETES**		
**OTHER**		
**FRAGILITY**		

PHARMACOLOGICAL HISTORY	
CHRONIC THERAPIES IN PLACE	
NOTES	

PHYSICAL EXAMINATION			
**WEIGHT**		**HEIGHT**	
**P.A. (mmHg)**		**F.C. (bpm)**	
**CARDIOVASCULAR**			
**PNEUMOLOGY**			
**NEUROLOGICAL**			
**PSYCHIATRIC**			
**OTHER**			

F O L L O W - U P				
	**DATE**	**IgG**	**Value**	**IgM**
**SEROLOGY**				
**SEROLOGY**				
**SEROLOGY**				
**TAKING CHARGE**	CARDIOLOGICAL ☐	PNEUMOLOGY ☐	NEUROLOGICAL ☐	PSYCHOLOGICAL
**OTHER**				

The Specialist Physician in Training

____

The Occupational Medical Director

____

POST-CoViD-19 RE-ENTRY

IES-6 Scale	
1. Since the beginning of the emergency, I thought about it when I didn't mean to	
2. Since the beginning of the COVID-19 emergency, other things kept making me think about it	
3. Since the beginning of the COVID-19 emergency, I felt watchful or on guard	
4. Since the beginning of the COVID-19 emergency, I tried not to think about it	
5. Since the beginning of the COVID-19 emergency, I was aware that I still had a lot of feelings about it, but I didn't deal with them	
6. Since the beginning of the COVID-19 emergency, I had trouble concentrating	

How afraid he was of the risk of	
1. Contracting SARS-CoV-2 / CoViD-19 infection	
2. Being quarantined	
3. Aggravating existing diseases	
4. Infecting family members	

1	Not at all	2	Partialy	3	Fairly	4	To a large extent	5	Extremely
