# Supplementary material for: COVID-19 Symptoms and Mental Health Outcomes among Italian Healthcare Workers: A Latent Class Analysis
Source: Healthcare (Basel). 2024 Jul 15;12(14):1403. doi: 10.3390/healthcare12141403 (PMC11275353; doi:10.3390/healthcare12141403)
Supplement: Supplementary file 1 [file healthcare-12-01403-s001.zip › Supplementary Materials survey ita v.pdf]

RIENTRO POST-CoViD-19

SCHEDA ANAGRAFICA E LAVORATIVA			
COGNOME		NOME	
MATRICOLA		GENERE	
CELLULARE		EMAIL	
MANSIONE		REPARTO	

RT-PCR SARS-CoV-2		
	**DATA**	**RISULTATO**
**RT-PCR**		
**RT-PCR**		
**RT-PCR**		
**RT-PCR**		

SORVEGLIANZA ATTIVA	
**CLASSIFICATO**	
**INDICE DI RISCHIO MdL (se classificato)**	
**CONTATTI EXTRA-LAVORATIVI**	

DECORSO CoViD-19			
**OSPEDALIZZAZIONE**			
**FEBBRE**		**ANOSMIA**	
**TOSSE**		**AGEUSIA**	
**DISPNEA**		**CONFUSIONE**	
**ASTENIA**		**VOMITO**	
**CEFALEA**		**DIARREA**	
**CONGIUNTIVITE**		**ASINTOMATICO**	
**DOLORI OSTEO-MUSCOLO-ARTICOLARI**		**ALTRO**	
**DURATA POSITIVITÀ**			
**NOTE / TERAPIA**			

POST CoViD-19			
**ASINTOMATICO**		**ANOSMIA**	
**TOSSE**		**AGEUSIA**	
**DISPNEA**		**CONFUSIONE**	
**ASTENIA**		**CONGIUNTIVITE**	
**CEFALEA**		**DOLORI OSTEO-MUSCOLO-ARTICOLARI**	
**NOTE / TERAPIA**			

RIENTRO POST-CoViD-19

ANAMNESI PATOLOGICA REMOTA		
**M. CARDIOVASCOLARI**		
**M. PNEUMOLOGICHE**		
**M. NEUROLOGICHE**		
**M. PSICHIATRICHE**		
**DIABETE**		
**ALTRO**		
**FRAGILITÀ**		

ANAMNESI FARMACOLOGICA	
TERAPIE CRONICHE IN ATTO	
NOTE	

ESAME OBIETTIVO			
**PESO**		**ALTEZZA**	
**P.A. (mmHg)**		**F.C. (bpm)**	
**CARDIOVASCOLARE**			
**PNEUMOLOGICO**			
**NEUROLOGICO**			
**PSICHIATRICO**			
**ALTRO**			

FOLLOW-UP				
	**DATA**	**IgG**	**Valore**	**IgM**
**SIEROLOGIA**				
**SIEROLOGIA**				
**SIEROLOGIA**				
**PRESA IN CARICO**	☐ CARDIOLOGICO	☐ PNEUMOLOGICO	☐ NEUROLOGICO	☐ PSICOLOGICO
**ALTRO**				

Il Medico specialista in formazione ☐ Il Dirigente Medico del Lavoro

________________________ ________________________

RIENTRO POST-CoViD-19

IES-6 Scale	
1. Dall'inizio dell'emergenza, tante cose mi fanno pensare al COVID-19	
2. Dall'inizio dell'emergenza COVID-19, ci penso anche senza volerlo	
3. Dall'inizio dell'emergenza COVID-19, mi sento nervoso e allarmato	
4. Dall'inizio dell'emergenza COVID-19, cerco di non pensarci	
5. Dall'inizio dell'emergenza COVID-19, mi rendo conto di avere ancora molte emozioni negative senza rendermene conto	
6. Dall'inizio dell'emergenza COVID-19, ho avuto difficoltà a concentrarmi	

Quanto ha avuto paura dal rischio di	
1. Contrarre l'infezione da SARS-CoV-2 / CoViD-19	
2. Essere messo in quarantena	
3. Aggravare malattie esistenti	
4. Contagiare familiari	

1	Per Niente	2	In Parte	3	Abbastanza	4	In buona Parte	5	Estremamente
